# Troponin Variants in Congenital Myopathies: How They Affect Skeletal Muscle Mechanics

**DOI:** 10.3390/ijms22179187

**Published:** 2021-08-25

**Authors:** Martijn van de Locht, Tamara C. Borsboom, Josine M. Winter, Coen A. C. Ottenheijm

**Affiliations:** Department of Physiology, Amsterdam Cardiovascular Sciences, Amsterdam UMC, Location VUmc, 1081 HZ Amsterdam, The Netherlands; m.locht@amsterdamumc.nl (M.v.d.L.); tamaraborsboom94@gmail.com (T.C.B.); jm.dewinter@amsterdamumc.nl (J.M.W.)

**Keywords:** troponin, skeletal muscle, sarcomere, congenital myopathy, contractility

## Abstract

The troponin complex is a key regulator of muscle contraction. Multiple variants in skeletal troponin encoding genes result in congenital myopathies. *TNNC2* has been implicated in a novel congenital myopathy, *TNNI2* and *TNNT3* in distal arthrogryposis (DA), and *TNNT1* and *TNNT3* in nemaline myopathy (NEM). Variants in skeletal troponin encoding genes compromise sarcomere function, e.g., by altering the Ca^2+^ sensitivity of force or by inducing atrophy. Several potential therapeutic strategies are available to counter the effects of variants, such as troponin activators, introduction of wild-type protein through AAV gene therapy, and myosin modulation to improve muscle contraction. The mechanisms underlying the pathophysiological effects of the variants in skeletal troponin encoding genes are incompletely understood. Furthermore, limited knowledge is available on the structure of skeletal troponin. This review focusses on the physiology of slow and fast skeletal troponin and the pathophysiology of reported variants in skeletal troponin encoding genes. A better understanding of the pathophysiological effects of these variants, together with enhanced knowledge regarding the structure of slow and fast skeletal troponin, will direct the development of treatment strategies.

## 1. Introduction

### 1.1. The Troponin Complex Regulates Muscle Contraction

**Figure 1:**
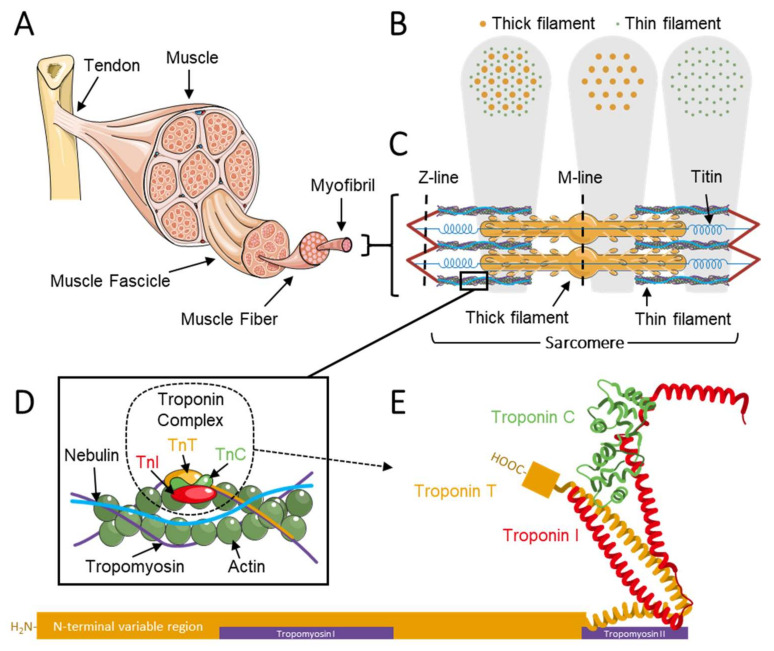
Schematic showing muscle structure at different levels of organization. (**A**) Whole muscle. (**B**) The thick and thin filaments are spaced in a symmetrical lattice. Every thick filament is surrounded by 6 thin filaments. The left panel shows a region of thick and thin filament overlap, the middle panel shows the H zone (including the thick filament and excluding the thin filament), and the right panel shows the I band (including the thin filament and excluding the thick filament). (**C**) The sarcomere. The grey shading indicates the corresponding lattice structure shown in (**B**). (**D**) The thin filament, including actin, nebulin, tropomyosin, and the troponin complex (troponin I, T, and C) (**E**) The troponin complex. Images A, C and D are modified from Servier Medical ART, licensed under a Creative Commons Attribution 3.0 generic license. (SMART—Servier Medical ART. https://smart.servier.com/, accessed on 20 July 2021).

Muscles are indispensable for daily life activities. Limb movement, respiratory function and the pumping motion of the heart all rely on the force generated by striated muscle. Striated muscle comprises skeletal muscle and cardiac muscle, the proteins in which share many of the same amino acid sequences but are encoded by different genes and have a different signal conduction pathway [1,2]. This review focuses on skeletal muscle troponin, a protein complex located on the thin filaments of sarcomeres (Figure 1A–C). The thin filament consists mainly of actin, tropomyosin, nebulin, and the troponin complex and regulates muscle contraction (Figure 1D) [3]. The thin filament has a molecular stoichiometry of 7:1:1, actin–tropomyosin–troponin, respectively [4,5]. The thick filament, mostly consisting of myosin, interacts with the thin filament in the sarcomere to generate force. Each thick filament is surrounded by six thin filaments in the shape of a hexagon (Figure 1B). Troponins are key players in the regulation of muscle contraction [6]. Upon binding of Ca^2+^ to troponin, conformational changes take place within the thin filament, exposing myosin binding sites on actin to enable cross-bridge formation [7,8,9].

The troponin complex is a 265 Å-long protein complex located along the thin filament on the F-actin–tropomyosin double helices with a ~38 nm periodicity (Figure 1D). The troponin complex is composed of three subunits: (1) the Ca^2+^-binding troponin C; (2) the inhibitory protein troponin I; and (3) the tropomyosin-binding and largest subunit, troponin T. The general structure of the troponin complex is comprised of two parts: (1) an approximately 46 kDa globular head or core domain consisting of troponin C, troponin I, and the C-terminal region of troponin T, and (2) a 160 Å-long rod-like tail consisting of the remainder of troponin T that interacts with the C-terminal region of tropomyosin (Figure 1E) [6,10]. When Ca^2+^ binds to troponin C, it changes the conformation of the troponin complex. Binding of TnC-Ca^2+^ to TnI inhibits TnI interaction with actin and tropomyosin [10]. Tropomyosin rotating along actin exposes the myosin binding sites on actin and enables attachment of myosin heads to the thin filament. The binding and release of myosin from actin is an ATP-dependent process termed the cross-bridge cycle and is the basis of striated muscle contraction [3,11,12,13,14,15].

Each troponin subunit is encoded by genes specific to muscle type, i.e., fast-twitch (type II) and slow-twitch (type I) (Table 1) [16,17,18,19,20]. These genes allow for a specialization of the muscle to serve its specific purpose All muscles contain a mixture of fiber types, but one fiber type can be predominant [21]. For example, in the extraocular muscles, fast-twitch fibers, which are needed for fast movements such as saccadic eye movements, are predominant. However, slow-twitch fibers are also required in extraocular muscles to enable gaze fixation [22,23]. Additionally, slow-twitch fibers are needed for posture regulation and movement and are therefore the predominant type in back muscles and muscles of the legs. Cardiac muscle troponins are encoded by cardiac-specific genes, with the exception of *TNNC1*, which encodes for slow skeletal and cardiac TnC [24,25]. The cardiac troponins exceed the scope of this review.

### 1.2. Troponin in Congenital Myopathies

In the past 30 years, variants in genes encoding for skeletal troponins have been identified to cause congenital myopathies, a group of heterogenetic disorders clinically characterized by skeletal muscle weakness and muscle atrophy [26,27,28,29,30,31]. Clinical features include a neonatal onset, a slow progressive nature, hypotonia, hypotrophy, delayed motor milestones, and respiratory dysfunction [28,32,33]. Although the precise epidemiology is still unknown, the incidence of congenital myopathies is estimated at approximately 1:25.000 [33,34]. Congenital myopathies present in five forms: nemaline myopathy (NEM), core myopathy, centronuclear myopathy, congenital fiber-type disproportion myopathy, and myosin storage myopathy [27,30,31,33,35,36].

How variants in genes encoding skeletal troponins lead to congenital myopathies and how they affect sarcomeric contractility is not completely understood. This lack of understanding impairs the development of treatment strategies. This review discusses the physiology of slow and fast skeletal troponin and how variants affect muscle contractility in congenital myopathies.

## 2. The (Patho)Physiology of Troponin C

**Figure 2:**
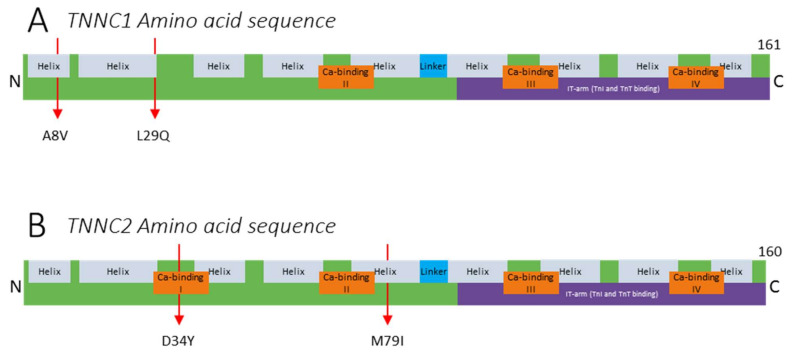
Protein structure and reported variants in (**A**) *TNNC1* (UniProt (P63316, TNNC1_Human)) and (**B**) *TNNC2* (UniProt (P02585, TNNC2_Human)).

TnC is the Ca^2+^-binding subunit of troponin and part of the EF-hand protein family [37,38]. The protein is encoded by two genes specific to muscle type: (1) *TNNC1*, which is located on chromosome 3p21.1 and encodes slow skeletal TnC containing 161 amino acids (UniProt (P63316, TNNC1_Human), Figure 2A); and (2) *TNNC2*, which is located on chromosome 20q12–q13.11 and encodes fast skeletal TnC containing 160 amino acids (UniProt (P02585, TNNC2_Human), Figure 2B) [39]. Both genes are highly conserved across species, which suggests all residues to carry significance for protein function and implies the importance of troponin C. TnC consists of four 12-amino-acid-long EF-hand motifs (I–IV) in two globular domains, the regulatory domain and the structural domain, which are connected by an α-helical linker (Figure 1E and Figure 2). The regulatory domain contains EF-hand motifs I and II and is located at the N-terminal side of the protein. During muscle contraction, the binding of Ca^2+^ to the Ca^2+^ binding sites of this domain is responsible for the conformational change in TnC [25]. This results in interaction of TnC with the switch region of TnI and contributes to the removal of adjacent inhibitory regions of TnI from myosin binding sites on actin [25]. While the regulatory domain of both ssTnC and fsTnC has two potential Ca^2+^ binding sites in EF-hand domains I and II, the Ca^2+^ binding site in EF-hand domain I in ssTnC is inactive because of the replacement of a key amino acid residue [40,41]. The Ca^2+^ binding site in EF-hand II is likely the trigger site of ssTnC, whereas its counterpart in EF-hand I modifies the characteristics of contraction [40,41,42,43,44,45]. The structural domain contains EF-hand motifs III and IV and is located at the C-terminal side of the protein. Both EF-hand motifs III and IV contain two high-affinity Ca^2+^-binding sites in both ssTnC and fsTnC, which are occupied by Ca^2+^ or Mg^2+^ under physiological conditions [25]. The incorporation of these two metal ions within the EF-hand helix–loop–helix motif stabilizes the structure of TnC [25,37]. Thus, binding of free intracellular Ca^2+^ to the Ca^2+^ binding sites in EF-hand motifs I and II initiates a cascade of events and conformational changes that eventually lead to muscle contraction.

### 2.1. Variants in TNNC1

Variants in *TNNC1* reported thus far predominantly cause hypertrophic cardiomyopathy (HCM) resulting from increased Ca^2+^ sensitivity of force of the cardiomyocytes [46,47]. Currently, no skeletal muscle dysfunction has been described in these patients. However, it is possible that a slow skeletal muscle phenotype is present but has not been observed. This could be due to the severity of the HCM phenotype, shadowing the effect on slow skeletal muscle function. Also, HCM limits patient mobility (e.g., due to shortness of breath), and therefore, weakness of slow skeletal muscles might be seen as a symptom of HCM. The majority of *TNNC1* variants are located in the EF-hands, with the exception of A8V, located near the N-terminus and leading to dilated cardiomyopathy, and L29Q, located at the putative interaction site for cTnI and therefore hindering the interaction between cTnI and cTnC, leading to hypertrophic cardiomyopathy (Figure 2A) [46,47,48,49]. Although the decreased TnI–TnC interaction might affect skeletal muscle function, this has not been investigated. Dilated cardiomyopathy (DCM) mouse and *Xenopus tropicalis* models have been studied, but no skeletal muscle defects have been reported [9,10,11,12,13,14,15,16,17,18,19,20,21,22,23,24,25,26,27,28,29,30,31,32,33,34,35,36,37,38,39,40,41].

### 2.2. Variants in TNNC2

Until recently, the gene encoding fast skeletal troponin C, *TNNC2*, was one of the last major genes encoding sarcomeric proteins that had not been implicated in muscle disease [30]. In 2021, van de Locht et al. identified two novel point variants in *TNNC2* in two unrelated families. [50] One family presented with the variant c.100C > A (p.Asp34Tyr; D34Y) located in the Ca^2+^ binding site I in the regulatory domain (Figure 2B). The other family presented with variant c.237G > C (p.Met79Ile; M79I) located close to the Ca^2+^ binding site II in the regulatory domain (Figure 2B). The patients presented with a distinct, dominantly inherited congenital myopathy, although with clinical improvement over time. This is atypical for congenital myopathy. Improvement might occur because of the switch from predominantly fast-twitch (*TNNC2*-expressing) myofibers in muscles after birth to a 50/50 distribution of slow- and fast-twitch myofibers at older age. [51] Molecular dynamics simulations indicated that regions responsible for Ca^2+^ binding and for binding of the TnI switch peptide (see below) are compromised. This was confirmed by studies of myofibers isolated from patients’ biopsies that revealed a reduced force response of the sarcomeres to submaximal [Ca^2+^], which contributes to muscle weakness. The pathogenicity of the variants was determined in experiments with myofibers in which the phenotype was [19] by replacing endogenous TnC in myofibers from healthy control subjects with recombinant, mutant TnC. Thus, pathogenic *TNNC2* variants occur and cause a mild congenital myopathy phenotype with patients surviving into adulthood. Screening of patients with congenital myopathy for variants in *TNNC2* is warranted. The lack of more reports on disease-causing variants in *TNNC1* and *TNNC2* underscores the importance of TnC for skeletal muscle structure and function.

## 3. The (Patho)Physiology of Troponin I

**Figure 3:**
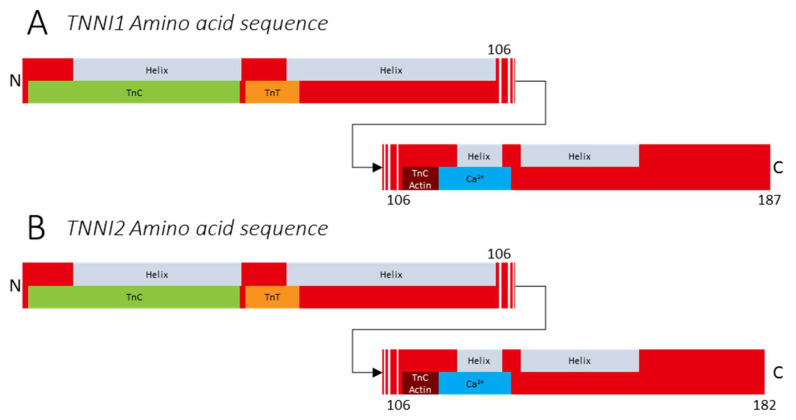
Protein structure of (**A**) *TNNI1* (UniProt (P19237, TNNI1_Human)) and (**B**) *TNNI2* (UniProt (P48788, TNNI2_Human)).

TnI is the inhibitory subunit of Tn [19]. The protein is encoded by three genes specific to muscle type: (1) *TNNI1* (UniProt (P19237, TNNI1_Human)), which is located on chromosome 1q32.1, encodes for slow skeletal troponin I (ssTnI), and consists of 187 amino acids from nine exons (Figure 3A); (2) *TNNI2* (UniProt (P48788, TNNI2_Human)), which is located on 11p15.5, encodes for fast skeletal troponin I, and consists of 182 amino acids from ten exons (Figure 3B); and (3) *TNNI3* (UniProt (P19429, TNNI3_Human)), which is located on 19q13.42, encodes for cardiac troponin I, and consists of 210 amino acids from eight exons. The 30 extra amino acids are located at the N-terminal side of the protein. As cardiac troponins exceed the scope of this review, we here focus on *TNNI1* and *TNNI2*. TnI and TnT are structurally connected in the troponin core domain with the IT arm, a rigid interface consisting of coiled-coil α-helices (Figure 1E and Figure 3). Importantly, TnI binds to actin polymers, inhibiting tropomyosin movement and thus preventing cross-bridge formation [52,53]. The inhibitory regions of TnI are highly conserved across species [54]. In the absence of Ca^2+^, TnI and TnC are weakly bound, and the C-terminal binding and inhibitory domains of TnI are bound to actin. This inhibits tropomyosin movement and myosin binding to actin. Possibly, the inhibitory effects of TnI are strengthened by interaction with tropomyosin [55]. However, to date the exact location of this tropomyosin interaction site on TnI is not mapped. As described above, binding of free Ca^2+^ to TnC induces a conformational change within the troponin complex, activating the TnI switch peptide. Subsequently, the TnI C-terminal binding domain binds to TnC instead of actin at the second domain, thereby enabling movement of tropomyosin and exposing the myosin binding sites on actin, which enables cross-bridge formation [52].

### 3.1. Variants in TNNI1

To date, *TNNI1* has not been implicated in disease. Like all troponin-encoding genes, *TNNI1* is highly conserved across species, suggesting all residues to carry high significance for protein function. During adulthood, troponin isoforms are mostly confined to a single muscle subtype. However, during development, a shift in TnI isoforms takes place. Using transgenic mice, Zhu et al. showed that at 9.5 days postconception, TnI isoforms are first expressed as ssTnI in newly formed myotubes, regardless of future fiber type [56]. Approximately 15 days postconception, fsTnI expression increases as secondary myotubes develop from primary myotubes. Furthermore, during development, ssTnI expression is higher in predominantly slow-twitch muscle (e.g., soleus) and fsTnI expression is higher in predominantly fast-twitch muscle (e.g., tibialis anterior). In humans, the developmental pattern of TnI expression has not been studied. However, it is likely that TnI expression shifts during development, but remains stable during adulthood, similar to its expression profile in mice [56]. Thus, the importance of ssTnI is indicated by the conservation of the gene across species and the expression pattern over time. It is likely that variants in *TNNI1* are lethal or lead to a severe phenotype at the embryonic or neonatal stage and therefore may not yet have been identified.

### 3.2. Variants in TNNI2

To date, *TNNI2* has not been implicated in congenital muscle disease, although it has been implicated in the skeletal muscle disorder distal arthrogryposis (DA) [57,58]. Bamshad et al. classified ten types of DA, and both DA1 and DA2B, also known as Sheldon–Hall syndrome, are caused by variants in *TNNI2* and *TNNT3*, respectively [59,60]. DA is an autosomal dominant disorder characterized by multiple congenital contractures of the distal limb joints [36,60,61,62,63,64,65]. The implication of fast skeletal troponins in DA has different possible explanations. (1) The disease origin could lie in muscle; it has been suggested that *TNNI2* and *TNNT3* variants that lead to DA cause an increased Ca^2+^ sensitivity in fast skeletal myofibers. This could result in increased muscle stiffness and therefore increased joint stiffness [66]. However, the mechanism underlying this hypercontractility needs further investigation. (2) Although troponins are considered sarcomeric proteins, *TNNI2* expression has been found in osteoblasts, chondrocytes of long bone growth plates, and cartilage [67,68,69]. The function of *TNNI2* expression in osteoblasts remains to be studied and the role of *TNNI2* in these tissues is still unclear. However, it might have implications for the distal limb joints. Zhu et al. suggested that DA could be caused by *TNNI2* variants that impair bone development [67].

## 4. The (Patho)Physiology of Troponin T

**Figure 4:**
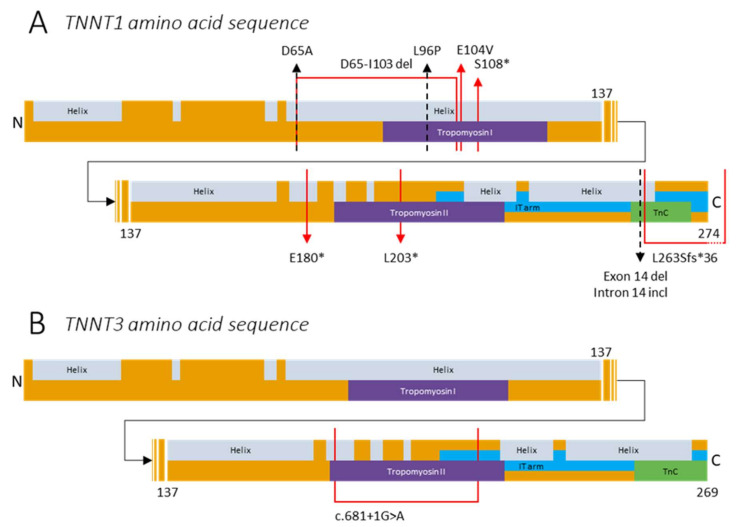
Protein structure and reported variants in (**A**) *TNNT1* (UniProt (P13805, TNNT1_Human)) and (**B**) *TNNT3* (UniProt (P45378, TNNT3_Human)).

TnT is the troponin subunit that anchors the troponin complex on tropomyosin [3,6,20,35,70,71,72,73]. TnT is encoded by three genes specific to muscle type: (1) *TNNT1*, which is located on chromosome 19q13.4 and encodes slow skeletal troponin T containing 278 amino acids (UniProt (P13805, TNNT1_Human), Figure 4A); (2) *TNNT2*, which is located on chromosome 1q32 and encodes cardiac troponin T containing 298 amino acids (UniProt (P45379, TNNT2_Human)); and (3) *TNNT3*, which is located on chromosome 11p15.5 and encodes fast skeletal troponin T (fsTnT) containing 269 amino acids (UniProt (P45378, TNNT3_Human), Figure 4B). Although *TNNT2* is mainly expressed in cardiac fibers, embryonic and neonatal skeletal muscles also express *TNNT2* [20,74,75,76,77]. Additionally, each gene produces several isoforms because of splicing events. Although TnT isoforms have a largely shared homology, they have slightly different effects on the Ca^2+^ sensitivity of the MgATPase, resulting in a different contractile response to Ca^2+^, because of phosphorylation and N-terminal charge differences [78]. Troponin T has an approximate axial length of 18 nm and a mainly structural role, and it can be divided into two structural domains: a rod-like tropomyosin-binding part and a globular regulatory part [79,80]. The globular region of TnT is part of the core domain of the troponin complex and contains the IT-arm, which connects TnT with TnC and TnI residues (Figure 1E and Figure 4) [52,80,81]. The IT-arm region of TnT is highly conserved and shows overlap between slow and fast skeletal troponin [82]. The rod-shaped N-terminal variable region of TnT is more structurally diverse between TnT genes. This region is mostly responsible for the differences between the slow and fast skeletal and cardiac isoforms and is regulated during development [20,83,84,85]. TnT comprises a T1 domain, spreading from the N-terminal side up to residue 158, and a T2 domain, which continues from residue 158 towards the C-terminal end [3,6]. Both domains have one tropomyosin-binding site, of which the T2 tropomyosin binding domain is Ca^2+^ dependent [52]. The T2 tropomyosin-binding domain is available for binding tropomyosin when Ca^2+^ is bound to TnC. The interaction of T2 and tropomyosin is thought to be stronger at lower Ca^2+^ concentrations [80].

### 4.1. Variants in TNNT1

Variants in *TNNT1* cause nemaline myopathy 5 (NEM5). Initially, NEM5 was confined to the Amish and therefore called Amish nemaline myopathy [29,61,71,72,86]. However, currently, multiple cases outside the Amish community have been reported. Generally, NEM5 has a severe phenotype, leading to (complete) loss of slow-twitch myofibers. Typically, patients do not survive past the age of two years, often because of respiratory failure [71,72,87,88]. *TNNT1* is one of at least 12 genes reported to be implicated in NEM, a congenital myopathy [29,72,89,90]. Hallmark features of NEM are skeletal muscle weakness and the presence of protein-dense inclusions known as nemaline bodies, or rods, in the myofibers [29,63]. NEM is a disease with a heterogeneous clinical phenotype and ranging severity [90].

In 2000, Johnston et al. were the first to describe a variant in ssTnT in the Old Order Amish community leading to NEM5. [71] Patients presented with the nonsense variant c.579G > T (p.Glu180*; E180*), resulting in truncation of ssTnT with 83 amino acids missing on the C-terminal side of the protein (Figure 4A). The truncated region contains the IT-arm region and the larger part of tropomyosin binding site 2. The truncation might impair the formation of the slow skeletal troponin complex and the localization of the troponin complex on the thin filament. Furthermore, it might lead to nonsense-mediated decay induced by the nonsense variant [91]. The ability of the truncated protein to form a complex with TnC and TnI has been studied with recombinant human fsTnT; studies have revealed a decreased binding efficiency of truncated fsTnT compared to wild-type fsTnT [92]. In line with these findings, Jin et al. found that the variant leads to a complete loss of ssTnT in NEM5 patients, as neither the truncated nor the intact TnT was present in muscle biopsies. As the expression of TnT isoforms is fiber-type specific, the absence of ssTnT leads to specific atrophy of slow-twitch myofibers and is suggested to be the molecular cause for this type of NEM [87].

The first case of NEM5 outside the Amish community was described by van der Pol et al. [72]. Two children presented with a heterozygous variant leading to skipping of exon 8 (c.309+1G > A; p.Asp65_Ile103del; Figure 4A). Both children had a postmortem diagnosis of NEM5 at the age of 3 and 5 years. Van der Pol et al. described a second patient in the same family presenting with skipping of exon 14 in addition to that of exon 8, resulting in NEM5 with neonatal symptoms and a progressive disease onset (Figure 4A) [72]. Exon 8 is part of tropomyosin binding site 1, and therefore, removal might alter ssTnT–tropomyosin binding. Additionally, removal of exon 14 might destabilize ssTnT and might reduce the affinity of ssTnT for the thin filament [93]. Another case of NEM5 outside the Amish community was reported by Marra et al. The patient presented with a homozygous nonsense variant c.323C > G (p.Ser108*; S108*) leading to a truncation of 155 amino acids in exon 9 (Figure 4A). This possibly impairs tropomyosin binding in tropomyosin binding site 1 [93,94]. Furthermore, Abdulhaq et al. described nine NEM5 patients from seven unrelated non-Amish families. [88] All patients had a homozygous variant c.574_577delinsTAGTGCT (L203*), resulting in a truncation on the C-terminal end of ssTnT (Figure 4A). The same variant was found in two patients by Fattahi et al. during clinical exome sequencing for neuromuscular diseases [95]. Konersman et al. described the first autosomal dominant case of NEM5 due to a heterozygous missense variant, c.311A > T (p.Glu104Val; E104V; Figure 4A) [89]. Family members of the proband were previously described as the first cases of hereditary NEM5 [96,97]. Recently, Fattori et al. described a new variant in an Italian patient presenting de novo heterozygous variant c.194A > C (p.(Asp65Ala); D65A; Figure 4A) [98]. In contrast to previously reported variants, this variant leads to a mild phenotype.

The *TNNT1* variants described above all result in truncation of ssTnT, which might impair the function of one or both of the tropomyosin binding sites, possibly resulting in an altered localization of the troponin complex relative to tropomyosin. Also, truncation of ssTnT could lead to impaired function of the troponin I and/or troponin C binding site(s), resulting in the inability to form the troponin complex. Furthermore, the absence of healthy and mutated ssTnT in slow-twitch fibers could result in atrophy of slow-twitch myofibers [71,87]. The other variants described in this study need further investigation to determine whether atrophy occurs in a similar fashion.

Recently, two novel recessive variants in *TNNT1* leading to congenital myopathy were described [99,100]. Pellerin et al. described three adults and one infant presenting with a missense homozygous variant c.287T > C (p.Leu96Pro; L96P; Figure 4A). [99] The variant substitutes the highly conserved leucine residue with proline, which is suggested to result in decreased binding affinity of ssTnT for tropomyosin. This could result in an inability of ssTnT to incorporate in the thin filament and therefore, undergo partial decay. All patients had a childhood onset and suffered from limb–girdle weakness with rigid spine and disabling contractures. Three patients required noninvasive mechanical ventilation. Multi-minicores and lobulated fibers were present in all patient muscle biopsies, and nemaline rods were present in half of the patient muscle biopsies. Petrucci et al. reported a 49-year-old female presenting with biallelic deletion c.786delG (p.Lys263Serfs*36; L263Sfs*36) in exon 13 [100]. The variant is predicted to cause modification of the last 16 amino acids and a small protein extension of ssTnT. The patient presented with a slowly progressive phenotype and suffered from myalgia, exercise intolerance, and dyspnea since infancy. Multi-minicores and nemaline rods were present in the patient’s muscle biopsy. Thus, recessive variants of *TNNT1* can manifest into a congenital myopathy.

### 4.2. Variants in TNNT3

Until recently, *TNNT3* was implicated only in DA, similar to *TNNI2* [36,57,62,101]. However, in 2018, Sandaradura et al. described a patient suffering from a phenotype that combined severe NEM and DA. [63] The patient had the variant c.681 + 1G > A, and cDNA data suggested that this donor splice site induced exon 14 skipping and increased levels of intron 14 preservation, contributing to a frameshift and premature stop codon or inclusion of ectopic sequences at the ssTnT C-terminus (Figure 4B). The patient was born at 31 weeks and was diagnosed with severe muscle weakness, hypotonia, contractures, high arched palate, hip dislocation, and thoracic scoliosis. During the neonatal period, invasive respiratory support was required. This was followed by noninvasive ventilation, which was sustained until death at the age of 8 months. Histochemical analysis of the muscle biopsy showed fast skeletal myofiber atrophy and nemaline rods confined to the fast skeletal myofibers. Additionally, hypertrophied slow skeletal myofibers were found with normal muscle structure. Furthermore, muscle tissue contained interstitial fibrosis and fiber degeneration without the presence of regenerating fibers. It was speculated that the NEM pathology in the patient resulted from a fsTnI deficiency in fast myofibers. During development, slow skeletal troponins are expressed before fsTn’s, and therefore, ssTnT might compensate for the lack of functional fsTnT [56]. Thus, the *TNNT3* variant c.681 + 1G > A results in exon 14 skipping, leading to a very severe phenotype of NEM and DA.

## 5. Therapeutic Strategies

Here, we focus on potential therapeutic strategies that directly target the sarcomere. For other therapeutic strategies, such as physical therapy, orthotic support, respiratory support, orthopedic surgery, and drug therapies that do not specifically target the sarcomere, but instead target downstream effects of variants in sarcomere genes, we refer to the reviews of Jungbluth et al. and Wang et al. [30,102].

### 5.1. Troponin Activators

Skeletal troponin variants often cause a decreased Ca^2+^ sensitivity of force in patients’ myofibers, i.e., a lower force production at submaximal Ca^2+^ levels. Particularly in patients with these variants, troponin activators might pose a therapeutic strategy [103,104,105].

To target slow skeletal myofibers, levosimendan, a slow-twitch/cardiac-specific troponin C activator, is available [106,107]. Levosimendan is approved for use in humans and has been developed to target cardiac myofibers. It exerts its effect through binding to slow skeletal/cardiac troponin C. As slow skeletal/cardiac troponin C is also the dominant troponin C isoform in slow-twitch skeletal myofibers, levosimendan might improve slow-twitch myofiber strength at submaximal levels of activation. Indeed, recent studies in human diaphragm, both in vitro and in vivo, indicated that exposure to levosimendan increases the Ca^2+^ sensitivity of force by 20–30% [108,109,110,111]. These studies illustrated the potential of levosimendan in improving myofiber function in myopathies. However, surprisingly, we observed no effect of levosimendan on the contractility of m. quadriceps myofibers of patients with NEM caused by variants in *NEB* (NEM2), who were shown to have a reduced Ca^2+^ sensitivity of force [112]. The myofibers studied expressed slow skeletal TnC, and the reason for the lack of an effect was unclear. Furthermore, the clinical relevance of levosimendan for patients with troponin variants might be limited, as no *TNNC1* or *TNNI1* variants leading to a skeletal muscle defect have been reported. In addition, variants in *TNNT1* lead to a lack of ssTnT protein, and importantly, levosimendan affects cardiac TnC, leading to undesired effects on cardiac function.

To target fast skeletal myofibers, *tirasemtiv* was used to repair the Ca^2+^ sensitivity of force in myofibers isolated from biopsies of patients with a *TNNC2* variant [50]. *Tirasemtiv* is a fast skeletal muscle troponin activator, which selectively increases Ca^2+^ sensitivity in fast-twitch myofibers. After treatment with *tirasemtiv*, the patients’ myofibers produced significantly more force at submaximal Ca^2+^ levels, restoring the Ca^2+^ sensitivity of force to levels of myofibers isolated from healthy subjects. De Winter et al. used *tirasemtiv*, in vivo and in vitro, to improve muscle performance in transgenic NEM mice by increasing the Ca^2+^ sensitivity of force and increasing fatigue tolerance [113]. However, *tirasemtiv* did not meet its primary endpoint in a recent phase 3 clinical trial in amyotrophic lateral sclerosis patients in part because of issues with tolerability (including dizziness, fatigue, nausea, weight loss, and insomnia) [114]. *Reldesemtiv* (formerly CK-2127107), a second generation fast skeletal muscle troponin activator that is structurally distinct from *tirasemtiv*, has a lower side effect profile and is currently under clinical trial investigation [115,116].

It is important to highlight that many patients with congenital myopathies, including those with variants in genes encoding skeletal troponin, experience difficulties with breathing due to impaired functioning of the respiratory muscles. During daily life activities, slow-twitch myofibers activated by small motor neuron cell bodies are recruited before fast-twitch myofibers activated by large motor neuron cell bodies are recruited (Henneman’s size principle) [117]. Thus, tidal breathing, during which predominantly slow-twitch myofibers are recruited, would benefit from slow troponin activation. However, during coughing, fast-twitch myofibers are recruited, which would benefit from fast troponin activation [118].

### 5.2. AAV Virus

Variants in genes encoding skeletal troponins cause a sequence of events, starting at the transcription of the mutated gene, that lead to translation of altered transcript and the generation of mutant protein and subsequently to muscle dysfunction in the form of myofiber atrophy or altered Ca^2+^ sensitivity of force. Future therapeutic approaches to combat skeletal troponin-related myopathies include the introduction of healthy skeletal troponin protein in patients’ muscles by adenoassociated viral vector (AAV) gene therapy. The introduced, healthy, protein competes with the mutated protein that causes the myopathy. Incorporation of the healthy protein could lead to significant improvement in muscle function, as shown by experiments in which endogenous TnC in myofibers isolated from patients with a *TNNC2* variant was replaced by recombinant, healthy TnC. This restored the Ca^2+^ sensitivity of force and thus increased force at submaximal Ca^2+^ levels [50]. Furthermore, the affinity of the healthy protein for binding partners might be higher compared to that of the mutated protein and thus result in better incorporation of the healthy protein [50]. Recent advances have shown the potential of AAV vectors in gene therapy for neuromuscular diseases, and AAV vector-mediated gene delivery was recently approved for spinal muscular atrophy [119,120,121,122]. Currently, trials with AAV delivery for treatment for muscular dystrophies are performed by intramuscular injections, isolated limb perfusion, or systemic IV injection [122]. However, these studies might be limited because of immune responses. Local administration could be effective when targeting affected muscles with particular functions, such as the diaphragm. Smith et al. found that intradiaphragmatic delivery of a recombinant AAV in IOPD patients significantly improved respiratory function during a phase I/II clinical trial (NCT00976352) [123]. However, intramuscular injection has limited transduction of the treatment around the area of injection.

### 5.3. Myosin Modulation

An alternative approach to combat muscle weakness due to skeletal troponin variants could be to target myosin and improve the efficiency of ATPase activity. To improve muscle contraction in *Acta1* His40Tyr knock-in mice, Lindqvist et al. cloned a cDNA construct encoding the cardiac alkali myosin light chain (MYL4) into an AAV expression plasmid and performed intramuscular injections of rAAV harboring a myosin transgene [124]. Introduction of the rAAV6:MYL4 increased the steady-state isometric maximal force-producing capacity of myofibers by 50% compared to mice injected with a control vector. The increase in force is likely the result of a greater strain per cross-bridge. Furthermore, they observed an increase in muscle weight after injection of rAAV6:MYL4 [124]. Thus, MYL4 is a potential future therapeutic strategy for NEM and troponin related myopathies.

The effect of Omecamtiv Mecarbil (formerly known as CK-1827452) on skeletal muscle was recently studied. Omecamtiv Mecarbil was originally developed to treat systolic heart failure [125]. It targets cardiac myosin (*MYH7*), which is also expressed in slow skeletal muscle, and thus, it could potentially increase muscle contractility of slow-twitch myofibers. Slow-twitch myofibers isolated from Neb cKO animals showed an increased Ca^2+^ sensitivity of force upon Omecamtiv Mecarbil administration [126]. However, Omecamtiv Mecarbil also binds to cardiac myosin molecules, possibly resulting in cardiac complications when Omecamtiv Mecarbil is administered to patients with a congenital myopathy.

## 6. Conclusions

**Figure 5:**
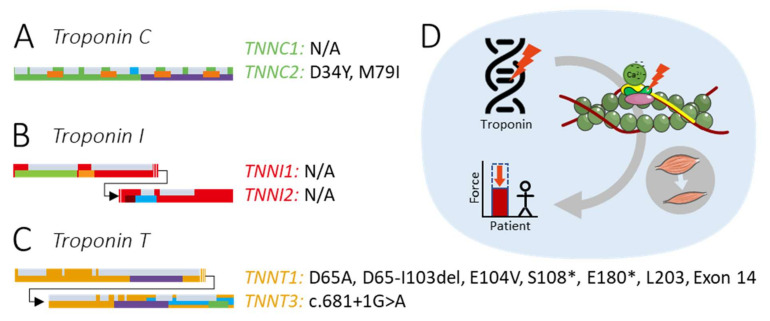
Summary figure. (**A**) Reported skeletal troponin C variants. (**B**) Reported skeletal troponin I variants. (**C**) Reported skeletal troponin T variants. (**D**) Variants in skeletal troponin-encoding genes cause a decrease of the Ca^2+^ sensitivity and/or atrophy of skeletal muscle, which result in muscle weakness. The image of the thin filament is modified from Servier Medical ART, licensed under a Creative Commons Attribution 3.0 generic license. (SMART—Servier Medical ART. https://smart.servier.com/, accessed on 20 July 2021).

Troponin is a key regulator of muscle contraction. To date, only a few variants in skeletal troponin genes have been reported (Figure 5A–C). This could be due to (1) the severity of the phenotype due to the indispensability of skeletal troponins, resulting in prenatal morbidity; (2) an absence of a phenotype, or (3) in the case of *TNNC1*, the skeletal muscle phenotype being overshadowed by a cardiomyopathy phenotype. Reported variants in skeletal troponin encoding genes predominantly lead to changes in the Ca^2+^ sensitivity of force, myofiber atrophy, and/or a lack of protein (Figure 5D). Several potential therapeutic strategies are available to counter the variant effects, such as troponin activators, introduction of exogenous protein though AAV gene therapy, and myosin modulation to improve muscle contraction. Research on these therapeutic strategies would greatly benefit from more data on the structure of skeletal troponin isoforms. With these data available, molecular dynamics modeling could help predict the structural and functional effects of variants and help solve the pathophysiology of muscle weakness caused by troponin variants.

## Figures and Tables

**Table 1:** Genes encoding troponin proteins and the abbreviations used.

Gene	Protein(s)	Abbreviation	Molecular Weight	Associated with
**TNNC1**	Slow skeletal troponin C Cardiac troponin C	ssTnC	18.4 kDa	Cardiomyopathy
**TNNC2**	Fast skeletal troponin C	fsTnC	18.1 kDa	Congenital myopathy
**TNNI1**	Slow skeletal troponin I	ssTnI	21.7 kDa	N/A
**TNNI2**	Fast skeletal troponin I	fsTnI	24.0 kDa	Distal arthrogryposis
**TNNI3**	Cardiac troponin I	cTnI	21.3 kDa	Cardiomyopathy
**TNNT1**	Slow skeletal troponin T	ssTnT	30.1 kDa	Nemaline myopathy
**TNNT2**	Cardiac troponin T	cTnT	35.9 kDa	Cardiomyopathy
**TNNT3**	Fast skeletal troponin T	fsTnT	31.8 kDa	Nemaline myopathy
